# Genetic mechanisms of axial patterning in *Apeltes quadracus*

**DOI:** 10.1093/evlett/qrae041

**Published:** 2024-08-07

**Authors:** Amy L Herbert, David Lee, Matthew J McCoy, Veronica C Behrens, Julia I Wucherpfennig, David M Kingsley

**Affiliations:** Department of Developmental Biology, Stanford University School of Medicine, Stanford, CA, United States; Howard Hughes Medical Institute, Stanford University School of Medicine, Stanford, CA, United States; Department of Developmental Biology, Stanford University School of Medicine, Stanford, CA, United States; Department of Pathology, Stanford University School of Medicine, Stanford, CA, United States; Department of Developmental Biology, Stanford University School of Medicine, Stanford, CA, United States; Department of Developmental Biology, Stanford University School of Medicine, Stanford, CA, United States; Department of Developmental Biology, Stanford University School of Medicine, Stanford, CA, United States; Howard Hughes Medical Institute, Stanford University School of Medicine, Stanford, CA, United States

**Keywords:** evolution, axial patterning, fourspine stickleback, QTL mapping, *HOX* genes, enhancers

## Abstract

The genetic mechanisms underlying striking axial patterning changes in wild species are still largely unknown. Previous studies have shown that *Apeltes quadracus* fish, commonly known as fourspine sticklebacks, have evolved multiple different axial patterns in wild populations. Here, we revisit classic locations in Nova Scotia, Canada, where both high-spined and low-spined morphs are particularly common. Using genetic crosses and quantitative trait locus (QTL) mapping, we examine the genetic architecture of wild differences in several axial patterning traits, including the number and length of prominent dorsal spines, the number of underlying median support bones (pterygiophores), and the number and ratio of abdominal and caudal vertebrae along the anterior–posterior body axis. Our studies identify a highly significant QTL on chromosome 6 that controls a substantial fraction of phenotypic variation in multiple dorsal spine and pterygiophore traits (~15%–30% variance explained). An additional smaller-effect QTL on chromosome 14 contributes to the lengths of both the last dorsal spine and anal spine (~9% variance explained). 1 or no QTL were detected for differences in the numbers of abdominal and caudal vertebrae. The major-effect patterning QTL on chromosome 6 is centered on the *HOXDB* gene cluster, where sequence changes in a noncoding axial regulatory enhancer have previously been associated with prominent dorsal spine differences in *Apeltes*. The QTL that have the largest effects on dorsal spine number and length traits map to different chromosomes in *Apeltes* and *Gasterosteus*, 2 distantly related stickleback genera. However, in both genera, the major-effect QTL for prominent skeletal changes in wild populations maps to linked clusters of powerful developmental control genes. This study, therefore, bolsters the body of evidence that regulatory changes in developmental gene clusters provide a common genetic mechanism for evolving major morphological changes in natural species.

The striking patterns of serially repeating structures found along the body axis of many animals have attracted the interest of philosophers and comparative anatomists for thousands of years (Aristotle, 350 BC(a),(b); Bateson, 1894; Owen, 1848). Some repeating features show low variation across groups, such as the characteristic number of seven cervical vertebrae found in most mammals (Flower, 1885). Other serial features vary widely between species and are often used for classification and naming. For example, fish groups show major differences in the total number of repeating units along the vertebral column, the ratio of vertebrae in the abdominal and caudal regions of the vertebral column, and the number, lengths, and positions of fins and spines along the dorsal midline (Mabee et al., 2002; Nelson et al., 2016). Many of these features have previously been linked to ecological adaptation. For example, the total number of vertebrae tends to increase with latitude in many fish groups (Jordan, 1891), a recurrent pattern that may be linked to altered swimming performance in colder waters (Reimchen & Cox, 2016). Similarly, the length and number of dorsal spines can serve as an important defense against predators in many animals, including arthropods, reptiles, dinosaurs, and fish (Gallina et al., 2019; Hoogland et al., 1956; Ramm et al., 2020; Shinohara & Takami, 2020).

In the Gasterosteidae family of stickleback fish, multiple genera are recognized and named for their contrasting patterns of serially repeating spine structures in the dorsal midline. For example, *Gasterosteus aculeatus* (“threespine stickleback”) typically have three dorsal spines, with the longest found in the middle of the series. *Apeltes quadracus* (“fourspine stickleback”) typically have four prominent dorsal spines, with the longest typically found at the anterior end of the series. In contrast, *Pungitius pungitius* (“ninespine stickleback”) have more numerous spines, all short in length and projecting to alternating sides of the dorsal midline (Woolton, 1984).

Although each stickleback group shows a typical number and pattern of axial structures, these patterns also show significant variation within each genus. For example, dorsal spine numbers can vary from one to seven in wild *A. quadracus* populations. Detailed sampling of over 500 populations in the maritime provinces of Canada has identified many populations where the predominant phenotype is three, four, or five dorsal spines (Blouw, 1982; Blouw & Hagen, 1984a). Higher spine number is correlated with predatory fish and the presence/absence of vegetation, covaries with increased spine number in other species, and confers a survival advantage against fish predation in both wild environments and laboratory studies (Blouw & Hagen, 1984b, 1984c, 1984d). Thus, dorsal spine variation in *Apeltes* is one of the best studied examples of major adaptive variation in repeating axial structures in vertebrates.

Although axial spine number differences in *Apeltes* show high heritability (Hagen & Blouw, 1983), the genetic architecture of the phenotype has never been studied by genome-wide approaches. Quantitative trait locus (QTL) mapping studies are well established in *G. aculeatus.* In threespine sticklebacks, genome-wide linkage studies have often revealed major loci that control 10%–70% or more of variance in classic evolutionary traits, including armor plate patterning, pelvic development, pigmentation, tooth number, and length of dorsal spines (Kingsley & Peichel, 2007; Peichel & Marques, 2017). However, other traits appear to be genetically complex, with no single locus contributing to more than 1% of the variance in the trait. Here, we use genome-wide QTL mapping in *A. quadracus* to characterize the genetic architecture of the striking spine number and spine length differences found in classic wild populations in Nova Scotia.

## Methods

### Ethical compliance

All ethical requirements were complied with during this study.

### Stickleback care

Wild sticklebacks were captured from locations around Nova Scotia, Canada, using minnow traps. Sticklebacks were treated in accordance with the Guide for the Care and Use of Laboratory Animals of the National Institutes of Health (NIH). Stickleback care at Stanford University was approved by the Institutional Animal Care and Use Committee (protocol no. 13834).

### QTL cross

A three-spine female from Railroad Crossing (“RRX,” 46.119 N, 60.485 W) and a five-spine male from Louisbourg Fortress (“Louisbourg,” 45.893 N, 59.999 W) were crossed in the field in Nova Scotia, Canada. The embryos were transported back to Stanford University and grown to adulthood. We then intercrossed a single adult F1 pair from the original field cross multiple times to generate 376 F2 progeny, which were grown to adulthood, euthanized, and fixed in 70% EtOH.

### Phenotyping

Lateral images of individual fish were taken using a Faxitron UltraFocus X-ray cabinet and processed using Fiji software (Schindelin et al., 2012). The following phenotypes were scored: spine number, dorsal and anal spine length, standard length, pterygiophore number, and abdominal and caudal vertebral count. Spine length measurements were taken using a segmented digital ruler to account for curvature. Abdominal vertebrae were distinguished by interaction with thin abdominal ribs. All other vertebrae were counted as caudal. Length measurements, including dorsal and anal spine lengths, were regressed against the standard length of individual fish using a custom Python script. A multivariate regression was also performed using sex as a covariate.

### NextRAD sequencing

NextRAD sequencing was performed by SNPsaurus. In brief, genomic DNA was converted into nextRAD genotyping-by-sequencing libraries (SNPsaurus, LLC) (Russello et al., 2015). Genomic DNA was first fragmented with Nextera reagent (Illumina, Inc.), which also ligates short adapter sequences to the ends of the fragments. The Nextera reaction was scaled for fragmenting 5 ng of genomic DNA, although 7.5 ng of genomic DNA was used for input to compensate for the amount of degraded DNA in the samples and to increase fragment sizes. Fragmented DNA was then amplified by polymerase chain reaction (PCR) for 26 cycles at 73 °C, with one of the primers matching the adapter and extending nine nucleotides into the genomic DNA with the selective sequence GTGTAGAGG. Thus, only fragments starting with a sequence that can be hybridized by the selective sequence of the primer will be efficiently amplified. The nextRAD libraries were sequenced on an Illumina HiSeq 4000 with one lane of 150 bp reads (University of Oregon).

The genotyping analysis used custom scripts (SNPsaurus, LLC) that trimmed the reads using bbduk (BBMap tools, http://sourceforge.net/projects/bbmap/): bbmap/bbduk.sh in=out= ktrim=r k=17 hdist=1 mink=8 ref=bbmap/resources/nextera.fa.gz minlen=100 ow=t qtrim=r trimq=10.

All reads were mapped to the reference genome with an alignment identity threshold of 0.96 using bbmap (BBMap tools). Genotype calling was done using callvariants (BBMap tools) (callvariants.shlist=ref_apeltes_rm.txt.align_samples out=apeltes_total.vcf ref=AP5.phap.1.fasta ploidy=2 multisample=t rarity=0.1 minallelefraction=0.1 usebias=f ow=t nopassdot=f minedistmax=5 minedist=5 minavgmapq=15 minreadmapq=15 minstrandratio=0.0 strandedcov=t). The resulting VCF file was filtered to remove alleles with a population frequency of less than 3%.

### Linkage map construction and QTL mapping

The program NGSEP (Tello et al., 2019) was used to convert the VCF file to a format suitable for JoinMap 5 (Van Ooijen, 2011). JoinMap 5 was then used to create a linkage map using the cross-pollinator population type (CP). The independence logarithm of the odds (LOD) value was set to 4, and only linkage groups with at least two markers were retained, resulting in 56 linkage groups. The genotyping data were extracted and formatted for use with the R package R/qtl version 1.66 (Broman et al., 2003). SNPLift version 1.0.4 (Normandeau et al., 2023) was used with default configuration and size of flanking regions set to 100 to convert genetic variant coordinates from the original *Apeltes* genome assembly used (AP5.phap.1) to a chromosome-level assembly (Liu et al., 2022) (GenBank: GCA_021346845.1; Assembly: UniBe_ApeQuad_1.0). Liftoff version 1.6.3 was used to convert Ensembl annotations (stickleback_v5_ensembl_genes.gff3, downloaded from https://stickleback.genetics.uga.edu/downloadData/) from version 5 of the *G. aculeatus* genome (stickleback_v5.0.1_assembly.fa, downloaded from https://stickleback.genetics.uga.edu/downloadData/) (Peichel et al., 2020) to the *Apeltes* genome using minimap2 parameters mm2_options=“-r 2k -z 5000” (Shumate & Salzberg, 2021). The R package R/qtl in R version 4.2.2 was used to generate QTL plots (Broman et al., 2003). A normal model was used for length measurements and a nonparametric model was used for count measurements. QTL mapping was performed both with and without sex as a covariate. However, the difference between these two analyses was negligible, and the final analysis does not include sex as a covariate. A total of 380 animals, including the two parents and grandparents, were phenotyped. A total of 269 markers were used for the initial QTL mapping. Two hundred seventy markers, including the peak marker in an axial enhancer region (referred to as *AxE*) (Wucherpfennig et al., 2022), were used for QTL mapping to identify the peak LOD score. Percent variance explained (PVE) was calculated using the following equation in R version 4.2.2: function(LOD, *N*) {100 * (1 − 10 ^ ((−2*LOD)/*N*))}.

### Genotyping the *HOXDB* locus

The following PCR primers were used to amplify the region containing *AxE*: forward primer (DK_ALH_255) 5ʹCAGCTGCTCTTAATGTCGCC3ʹ, reverse primer (DK_ALH_256) 5ʹCAAAGAGGGAGAGCAGACGG3ʹ. 2X Phusion Green Hot Start II High-Fidelity PCR Master Mix (ThermoFisher Scientific, F566) was used to amplify the 427 bp PCR product with the following thermocycler settings: 98 °C (3 min), 35 cycles of 98 °C (10 s)/60 °C (30 s)/72 °C (10 s) and a final extension of 72 °C (10 min). Five microliters of PCR product was then digested with NdeI (catalogue no. FD0583; New England Biolabs) and run on a 2% agarose gel to score cut and uncut bands. PCR products were purified using a QIAquick PCR purification kit (Qiagen, 28106) and quantified using a Qubit DNA High Sensitivity Kit (Invitrogen, Q32854). Samples were sent for Amplicon-EZ sequencing at Azenta Life Sciences (2 × 250 bp). FastQC (Andrews, 2010) was used to perform quality control checks on the raw Illumina reads, and Trimmomatic version 0.36 (Bolger et al., 2014) was used to remove adapters and nucleotides with poor quality. Next, if the forward and reverse reads overlapped, paired sequence reads were merged to form a single read. These merged reads were then aligned to a reference sequence matching the expected PCR product, and variant detection was performed using AZENTA’s Amplicon-EZ program.

## Results

### A QTL cross in *A. quadracus*

**Figure 1.**
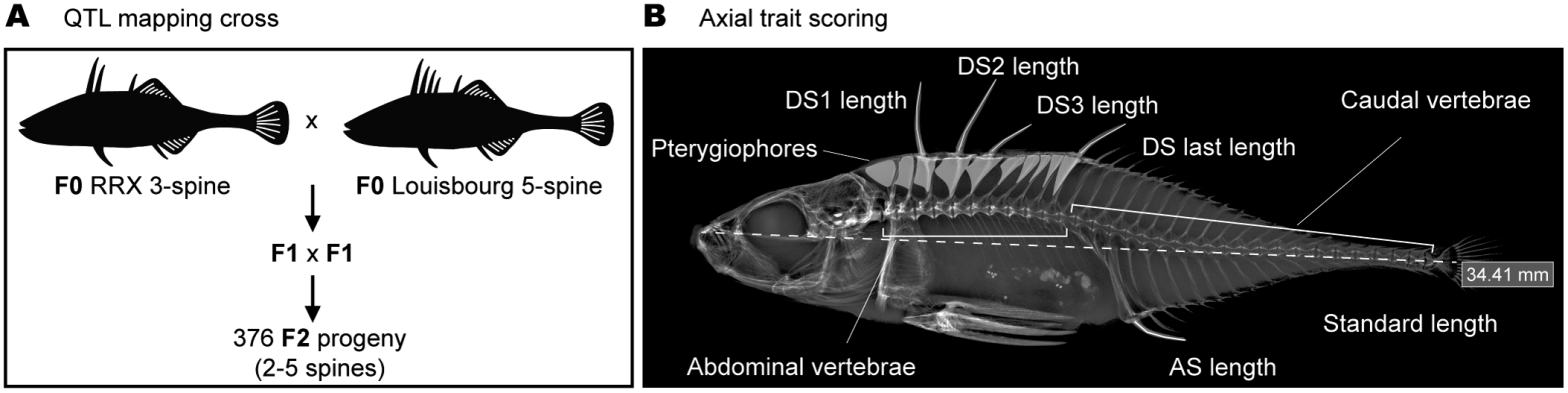
Quantitative trait locus (QTL) mapping of axial patterning traits in *Apeltes quadracus.* (A) A schematic showing the QTL crossing scheme in which a three-spine *Apeltes* was crossed to a five-spine *Apeltes*. The F1 generation was raised to adulthood and intercrossed to produce 376 F2 adult animals. (B) Axial traits were scored in adult animals using X-ray analysis. Traits scored included dorsal spine (DS) and anal spine (AS) length, dorsal spine and pterygiophore number, and abdominal and caudal vertebral number. Length measurements were regressed against the standard length of the animal.

In May 2018, we collected *A. quadracus* at various locations around Nova Scotia, Canada, using previous studies as a guide to identify sites with substantial dorsal spine variation (Blouw & Hagen, 1984b, 1984c, 1984d). A three-spined *Apeltes* female from a location with a high prevalence of low-spined fish (Railroad Crossing or RRX) was crossed in the field to a five-spined *Apeltes* from a location with a high prevalence of high-spined fish (Louisbourg Fortress or Louisbourg). The F1 embryos were transported to Stanford University, where they were grown to adulthood (Figure 1). We found that *Apeltes* reproductive behavior in the lab closely resembled what has been reported previously in the wild (Rowland, 1974). In the tank, adult *Apeltes* males built cup-shaped nests in plastic aquarium plants using a glue-like substance secreted from their kidney, and repeatedly oxygenated fertilized eggs by pumping water through their operculum directly into the nest (Supplementary Movie 1) (De Ruiter & Bonga, 1985). In some instances, we observed males building nests on airstones, thereby precluding the necessity of oxygenating embryos (Supplementary Movie 2). Although *Apeltes* females typically produce smaller egg clutches compared to *Gasterosteus*, we were able to establish a natural crossing scheme in which a single F1 male and female were kept together in a tank. Whenever we observed nests with fertilized eggs, the entire nest was carefully collected. Males would subsequently rebuild nests and reproduction would recommence. In total, 759 F2 embryos were collected from a single F1 mating pair using sequential clutch collections over a span of ~3.5 months.

A total of 376 adult F2 fish were raised to adulthood, and these fish plus the two F1 parents and the two F0 grandparents, were scored for the following axial traits using X-ray analysis: number of dorsal spines, lengths of each dorsal spine, number of pterygiophore bones in the dorsal midline (which can occur either with or without dorsal spines), number of abdominal vertebrae, number of caudal vertebrae, and length of the anal spine on the ventral side of the fish. All spine length measurements were standardized by regressing against standard lengths. Genotype data for the grandparents, parents, and all 376 F2 fish were obtained using nextRAD sequencing.

### Mapping of *Apeltes* axial traits

**Figure 2.**
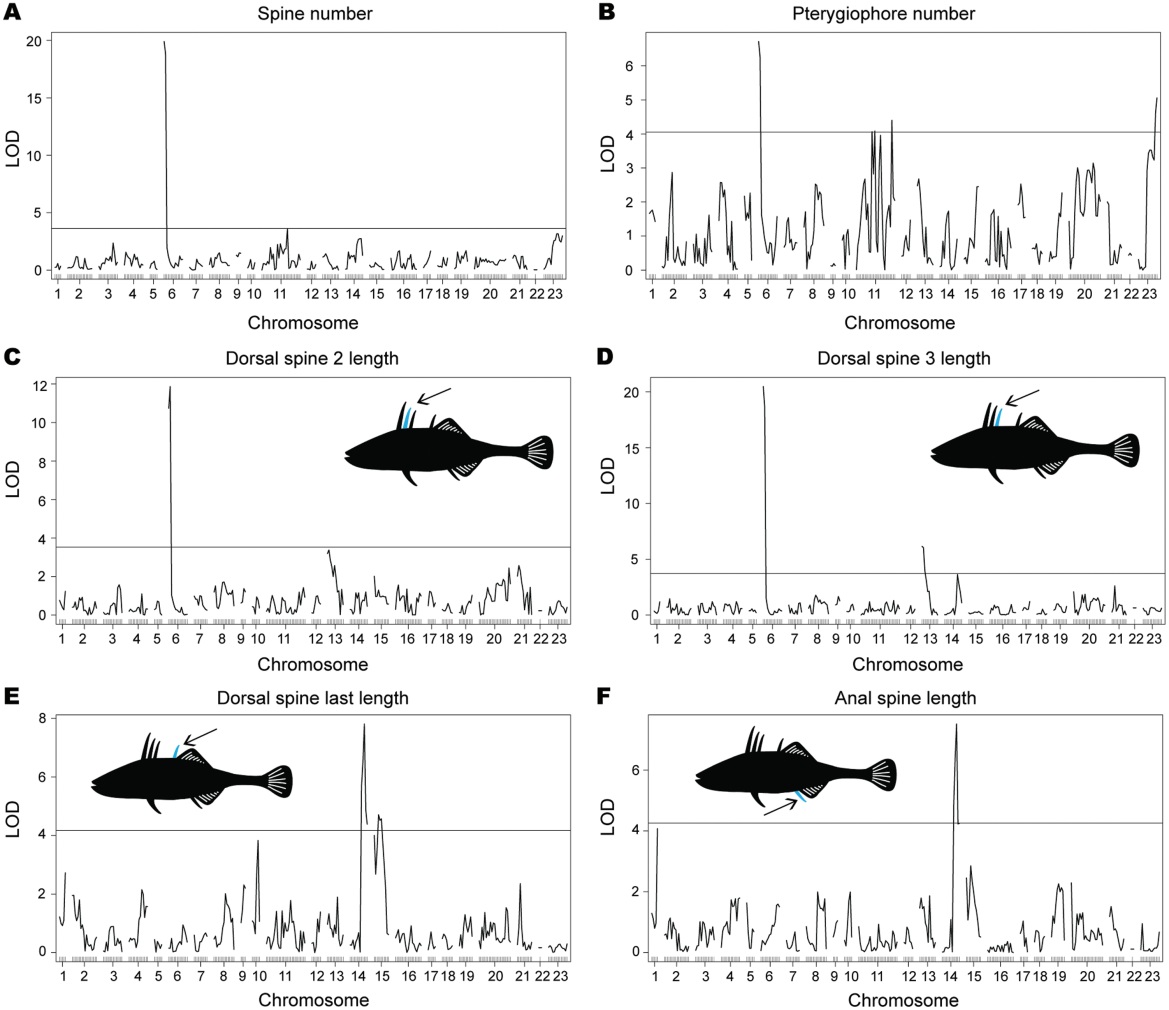
Axial traits map to chromosomes 6 and 14. (A–D) A series of axial traits, including spine number, pterygiophore number, and dorsal spine 2 and dorsal spine 3 lengths, all mapped to *Apeltes* chromosome 6. (E, F) The last dorsal spine length and anal spine length both mapped to chromosome 14. The horizontal line in the graph is indicative of genome-wide significance and is obtained with the threshold set at 1,000 permutations of the data and α = 0.05.

Using QTL analysis, a suite of axial patterning traits, including spine number, pterygiophore number, and lengths of dorsal spines 2 and 3, all mapped to *Apeltes* chromosome 6 (Figure 2). This is the same chromosomal region harboring the *HOXDB* enhancer (the *AxE*), that we previously showed plays a minor role in both spine number and dorsal spine 2 length in *Gasterosteus* (Wucherpfennig et al., 2022). In the previous study, we also used candidate gene association mapping to demonstrate that spine number in two wild *Apeltes* populations and dorsal spine 3 length in one *Apeltes* population were associated with genotypes in the *HOXDB* region, the only locus that was tested (Wucherpfennig et al., 2022). The peak marker for the *Apeltes* association mapping was a dinucleotide change, from GG to AA in *AxE*, which led to increases in both spine number and dorsal spine 3 length in several *Apeltes* populations. In our current genome-wide linkage analysis, we found that an initial peak QTL marker on chromosome 6 was located approximately 1 Mb from the *AxE* region, indicating that the *HOXDB* cluster was likely important for the axial trait differences segregating in the cross (also see below).

We previously determined in *Gasterosteus* that the genomic region associated with spine number variation was not significantly associated with vertebral differences, suggesting a particular role for spine patterning rather than axial patterning in general (Wucherpfennig et al., 2022). To test this in *Apeltes*, we counted abdominal and caudal vertebral numbers and did not find a significant QTL peak on the same region of chromosome 6. Overall, we found no significant QTL peaks associated with abdominal vertebral number and identified only a minor peak that barely reached significance with a peak LOD score of 3.98 for caudal vertebral number on chromosome 17 (Table 1 and Supplementary Figure 1). Overall, this suggests that in *Apeltes* as in *Gasterosteus*, the role of *AxE* and *HOXDB* appears limited to spines. We similarly observed no QTL peak for dorsal spine 1 (Supplementary Figure 1), confirming that the chromosome 6 region is primarily involved in a particular set of posterior spines as shown by expression analysis performed previously (Wucherpfennig et al., 2022).

**Table 1.** Logarithm of the odds (LOD) and percent variance explained (PVE) values for traits with significant quantitative trait locus peaks. The marker with the highest LOD score (peak marker) for spine number, pterygiophore number, and dorsal spine 2 and 3 lengths was located in the axial enhancer of the *HOXDB* locus and explained between 10% and 29% of the variance for these traits. Peak markers on chromosome 14 and 17 explained ~9% and ~5% of variance in the length of the last dorsal and anal spines, or caudal vertebral number, respectively.

Trait	Peak marker	LOD score	PVE (%)
Spine number	chr6_AxE peak	22.07	23.47
Pterygiophore number	chr6_AxE peak	9.29	10.65
Dorsal spine 2 length	chr6_AxE peak	14.33	15.98
Dorsal spine 3 length	chr6_AxE peak	27.59	29
Dorsal spine last length	chr14_12137166	7.8	9.05
Anal spine length	chr14_12137166	7.52	8.73
Caudal vertebral number	chr17_15773417	3.98	4.70

Finally, while several key axial traits mapped to the *HOXDB* enhancer of chromosome 6, we also identified two traits (length of the last dorsal spine and anal spine length) that mapped to a region of chromosome 14, which has not been previously shown to be involved in spine length phenotypes in any stickleback species (Figure 2E and F). The peak marker for the length of both the last dorsal spine and the anal spine was located in an intron of the gene *Deleted in Colorectal Cancer* (*DCC*), which is a *Netrin-1* receptor (Meijers et al., 2020).

### Genotyping the *HOXDB* enhancer

**Figure 3.**
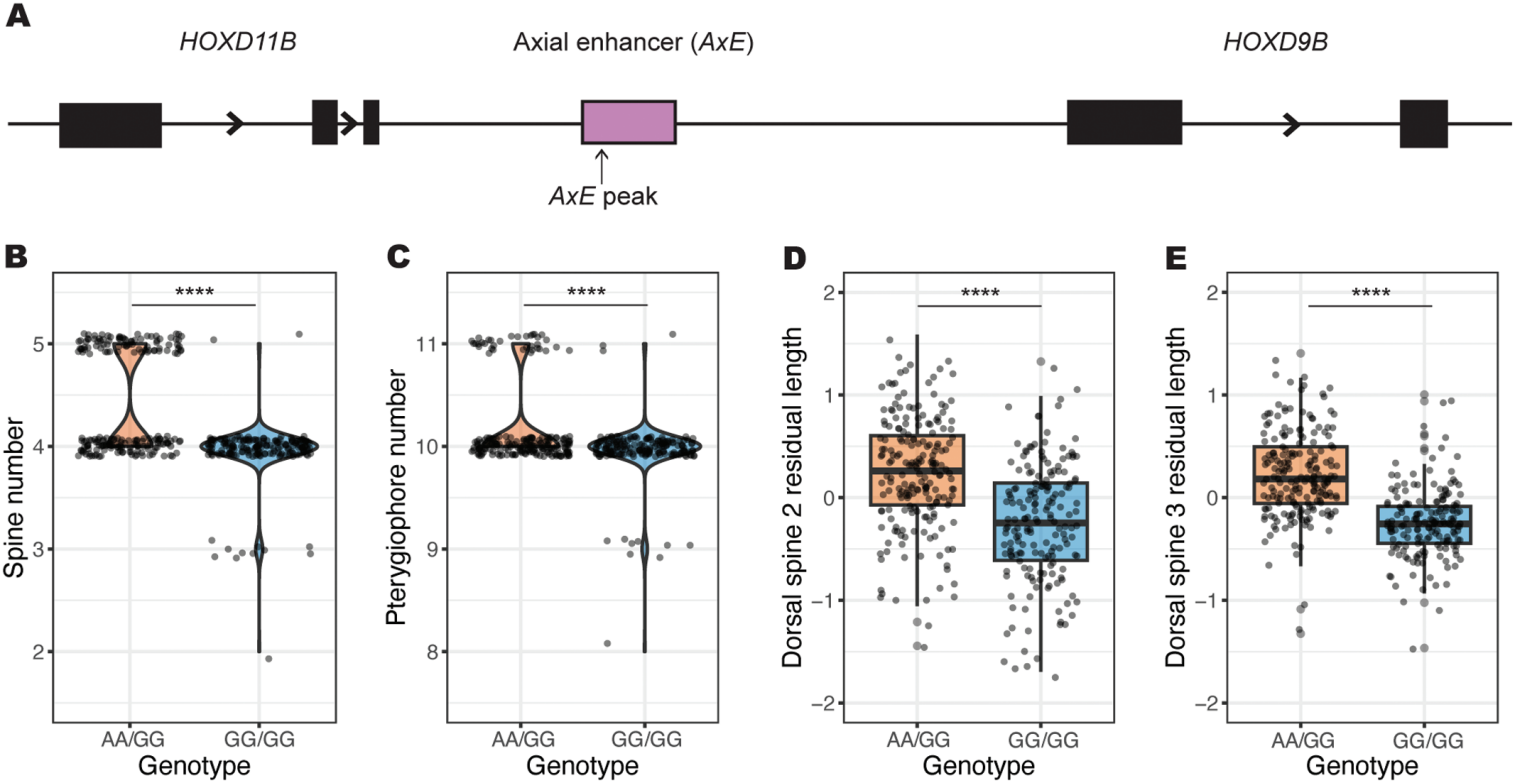
Phenotypic shifts based on genotypes at the *HOXDB* locus. (A) The axial enhancer (*AxE*) is located between the genes *HOXD11B* and *HOXD9B*, and includes a two-base pair dinucleotide change that segregates in wild *Apeltes* populations (*AxE* peak arrow) (Wucherpfennig et al., 2022). (B–E) Presence of the AA allele at *AxE* leads to significant phenotypic shifts towards higher spine numbers, higher pterygiophore numbers, and longer dorsal spine 2 and dorsal spine 3 lengths in F2 progeny. Low-spine number was scored as two to four spines while high-spine number was scored as five spines. Animals with low pterygiophore number had 8–10 pterygiophores while high pterygiophore animals had 11 pterygiophores. Spine length measurements were regressed against the standard length of individual fish. Data points were jittered in the x- and y-axes to prevent overplotting. Exact *p*-values: *p* < 2.2e-16 (B, spine number, Fisher’s exact test), *p* = 5.858e-08 (C, pterygiophore number, Fisher’s exact test), *p* = 9.52e-16 (D, dorsal spine 2 length, two-sample *t*-test), and *p* = 2.2e-16 (E, dorsal spine 3 length, two-sample *t*-test).

To determine if the same dinucleotide shift in *AxE* identified through association mapping was involved in the region linked to axial trait differences in our QTL cross, we genotyped all 376 F2 animals, as well as the F1 parents and F0 grandparents for this sequence change. From this, we identified a significant correlation between genotype and phenotype at the *AxE* locus (Figure 3). All F2 animals generated in the QTL cross were either heterozygous for the high-spine allele (AA/GG) or homozygous for the low-spine allele (GG/GG) at *AxE*, which was confirmed through sequencing. Animals with one copy of the high-spine allele (AA/GG) had statistically significantly higher numbers of spines and pterygiophores, as well as longer dorsal spine 2 and 3 length. Animals homozygous for the low-spine allele (GG/GG) exhibited fewer spines and pterygiophores and shorter spine length (Figure 3). When we incorporated the genotyping results for the *AxE* peak marker into our QTL analysis, we found that this location had the highest LOD score for these axial traits and explained between ~10% and 30% of the variance, depending on the trait (Table 1). Interestingly, previous association mapping identified homozygous high-spine alleles within the population (AA). This difference highlights the importance of *AxE* in axial trait development, since a single allele can contribute to higher spine and pterygiophore numbers and increased spine lengths. Moreover, we identified a previously unreported 30 bp deletion within *AxE* specifically in the low-spine population, which removes some sequences otherwise conserved between sticklebacks and other more distantly related percomorph fish outgroups, such as *Medaka* and *Tetraodon* (Supplementary Figure 2). This additional variation may play a role in the significant axial trait differences observed between the high- and low-spine populations.

## Discussion

The evolution of the vast array of body plans seen in nature has long been a source of fascination for biologists. Here, we used an unbiased QTL mapping approach to identify regions of the genome involved in axial trait patterning in wild populations of *A. quadracus*. We found that two axial traits, the last dorsal spine and anal spine lengths, map to a region on chromosome 14, with the peak marker residing in an intron of the *Netrin-1* receptor *DCC.* Notably, this region has not previously been associated with axial traits in any stickleback species. While *Netrin-1* is well known for its role in axon guidance in the nervous system, it also contributes to bone development by promoting the differentiation of osteoclasts (Mediero et al., 2015). In humans, the *DCC* gene is known for its role as a tumor suppressor involved in cancer, with reduction or loss of *DCC* expression found in the majority of colorectal cancers (Mazelin et al., 2004). Importantly, *DCC* can regulate apoptosis through a binding mechanism whereby downstream cell death pathways are activated unless *DCC* is bound by its ligand *Netrin-1* (Mazelin et al., 2004). Further fine-scale mapping studies will be necessary to test the significance of the *DCC* intronic region, or other linked regions on chromosome 14, in driving axial trait differences in wild *Apeltes*. It will also be interesting in the future to explore a potential role for *Netrin-1* and *DCC* signaling in spine growth, perhaps through driving (or reducing) cell growth capacity via regulation of osteoclast differentiation or apoptosis.

We also determined that multiple aspects of axial patterning in wild *Apeltes* fish, including spine number, the length of dorsal spines 2 and 3, and the number of pterygiophores, are controlled by a major genetic locus on chromosome 6. The substantial PVE by this QTL rank in the top 10% of all genetic effects ever reported in sticklebacks (Peichel & Marques, 2017). Typical genetic crosses may overestimate the magnitude of the phenotypic effect attributable to individual QTL (Slate, 2013). However, the large effect size of the chromosome 6 region has now been confirmed by two independent methods: genome-wide linkage in laboratory F2 crosses (this study) and a single-locus candidate association study in wild fish (Wucherpfennig et al., 2022). In both lab crosses and wild populations, the genotype at the *HOXDB* locus explains between ~15% and 30% of the variance in dorsal spine phenotypes.

This major-effect axial patterning QTL on chromosome 6 is likely due to regulatory changes in the *HOXDB* locus. This locus contains three clustered homeodomain transcription factor genes (*HOXD4B*, *HOXD9B*, and *HOXD11B*), and the intergenic region between *HOXD9B* and *HOXD11B* corresponds to the peak genetic association in previous high-resolution mapping studies of spine number in wild *Apeltes* populations in Nova Scotia (Wucherpfennig et al., 2022). A noncoding *AxE* sequence maps within this interval and can drive expression at particular anterior–posterior levels during larval development. Previous studies have shown this enhancer has two adjacent single-nucleotide polymorphism (SNP) changes in high-spine fish (five- and six-spined) versus low-spine fish (three- and four-spined) populations in Louisbourg. While genome editing of specific DNA bases has not yet been established in *Apeltes*, induced deletions in either the *AxE* enhancer or the *HOXDB* coding regions of *Gasterosteus* can alter the length of dorsal spines and pterygiophore numbers in sticklebacks, confirming a key functional role of the *HOXDB* locus in stickleback axial patterning (Wucherpfennig et al., 2022).

*Hox* genes are known for their important role in assigning distinct developmental fates to elements in repeating series, such as body segments in insects and skeletal morphologies in the vertebral column and limb bones of vertebrates (Averof & Patel, 1997; Burke et al., 1995; Carroll, 1995). Laboratory mutations in *Hox* genes can cause dramatic transformations of body parts and segmental identity but are usually associated with decreased viability or infertility. This led to the hypothesis that *cis*-regulatory elements of *Hox* genes would be more likely to provide a suitable genomic substrate for evolutionary change in wild populations (Akam, 1998; Stern, 1998). A handful of cases are now known where significant phenotypic differences in wild species have been mapped to natural variation in *Hox* loci (Kingsley et al., 2024; Stern, 1998; Tian et al., 2019; Wucherpfennig et al., 2022). *Apeltes* now provides one of the best examples in vertebrates for natural regulatory variation in *Hox* genes contributing to major changes in the axial skeletal patterns of wild species.

Extensive previous environmental, co-variation, and experimental predation studies suggest that spine number variation in *Apeltes* is an adaptive trait that likely varies with the intensity of insect, bird, and fish predation in different environments (Blouw & Hagen, 1981, 1984a, 1984b, 1984c, 1984d). Interestingly, alternative spine morphs are maintained in *Apeltes* populations for extended periods of time (Blouw & Hagen, 1981). For example, we found higher-spined and lower-spined *Apeltes* morphs in almost 50:50 ratios at the Louisbourg site in 2019 (Wucherpfennig et al., 2022), and neither morph has swept to fixation in the 40 years since both forms were first reported in this area in earlier collections (Blouw & Hagen, 1984d). Such axial patterning traits may be maintained over time because different morphs have contrasting advantages in the presence of fish or bird predation, and predators and other environmental factors may themselves vary seasonally and from year to year.

The genetics of major anterior–posterior patterning traits in *Apeltes* show interesting similarities and differences with the genetic architecture of related traits in the distantly related *Gasterosteus* genus, which diverged from *Apeltes* over 16 million years ago (Kawahara et al., 2009; Mattern, 2006). In both *Apeltes* and *Gasterosteus*, genetic variation at the *HOXDB* locus contributes to dorsal spine variation, likely through independent regulatory mutations in the same *AxE* axial patterning enhancer (Wucherpfennig et al., 2022). However, what appears to be a major-effect locus for dorsal spine patterning in *Apeltes* (up to 30% PVE) is only a small-effect locus in *Gasterosteus* (6%–8% PVE). Conversely, *Gasterosteus* shows a different major-effect locus on chromosome 4 that controls many different aspects of defensive armor and behavior, including the length of spines, the number of armor plates, and the pattern of sensory neuromasts along the sides of fish (20%–80% PVE) (Archambeault et al., 2020; Colosimo et al., 2004; Cresko et al., 2004; Greenwood et al., 2016; Howes et al., 2017; Roberts Kingman et al., 2021). This major-effect locus in *Gasterosteus* appears to be an evolved supergene region with significant effects coming from multiple linked developmental control genes, including two secreted signaling molecules (*Eda* and *Stanniocalcin2a*), and one homeodomain transcription factor (*Msx2a*) (Archambeault et al., 2020; Howes et al., 2017; O’Brown et al., 2015; Roberts Kingman et al., 2021). *Eda*, *Stanniocalcin2a*, and *Msx2a* all map to chromosome 4 in *Apeltes*, but this region does not have a measurable effect on the various axial traits measured in this study.

Why might different chromosome regions evolve to be large effect loci controlling prominent axial patterning traits in the two different stickleback groups? Marine *Gasterosteus* fish have a complete set of armor plates along their lateral sides, can frequently migrate between salt and freshwater environments, and have a Robertsonian fusion associated with reduced recombination in the *Eda–Msx2a–Stc2a* region (Liu et al., 2022). In contrast, *Apeltes* fish lack armor plates, have a more restricted species range in brackish environments, and lack the Robertsonian fusion that occurred on chromosome 4 in *Gasterosteus*. We speculate that substantial differences in skeletal anatomy, migratory lifestyle, and chromosome karyotypes may contribute to which genes have evolved into large effect loci in the two different species.

Although the QTL regions of largest effect differ between *Apeltes* and *Gasterosteus*, in both cases, the major loci map to a cluster composed of linked powerful developmental control genes. For *Gasterosteus*, the individual genes in the chromosome 4 supergene region are structurally unrelated to each other (*Eda*, *Msx2a*, *Stc2a*). High-resolution mapping studies suggest that each of these genes have undergone separate *cis*-acting regulatory changes that contribute to the overall morphological effects of the major chromosome 4 QTL region (Archambeault et al., 2020; Howes et al., 2017; O’Brown et al., 2015; Roberts Kingman et al., 2021). In *Apeltes*, the individual genes in the chromosome 6 region are all structurally related homeodomain transcription factors (*HOXD4B*, *HOXD9B*, and *HOXD11B*). All three of these closely linked *HOXDB* genes show significant *cis*-acting expression changes in high- and low-spined morphs of sticklebacks (Wucherpfennig et al., 2022). Future work will examine whether the multiple expression differences can all be traced to DNA changes in the same embedded *AxE* enhancer, or whether multiple independent regulatory changes may have occurred in the larger *HOXDB* region. Further development of genetic and genomic editing tools in *Apeltes* should help illuminate the role of individual DNA changes in evolving new axial patterns in wild species, and the role of clustered developmental control genes in producing major changes in skeletal morphology in vertebrates.

## Supplementary material

Supplementary material is available online at *Evolution Letters*.

qrae041_suppl_Supplementary_Figures_1-2

## Data Availability

The sequencing data generated from this study are available at the NCBI Sequence Read Archive under project number PRJNA1134107. The data and code used in the project are available on Figshare (https://doi.org/10.6084/m9.figshare.26221529).
