# Supplementary material for: Genetic mechanisms of axial patterning in Apeltes quadracus
Source: Evol Lett. 2024 Aug 7;8(6):893–901. doi: 10.1093/evlett/qrae041 (PMC11637603; doi:10.1093/evlett/qrae041)
Supplement: qrae041_suppl_Supplementary_Figures_1-2 [file qrae041_suppl_supplementary_figures_1-2.zip › Final_supplemental_materials.pdf]

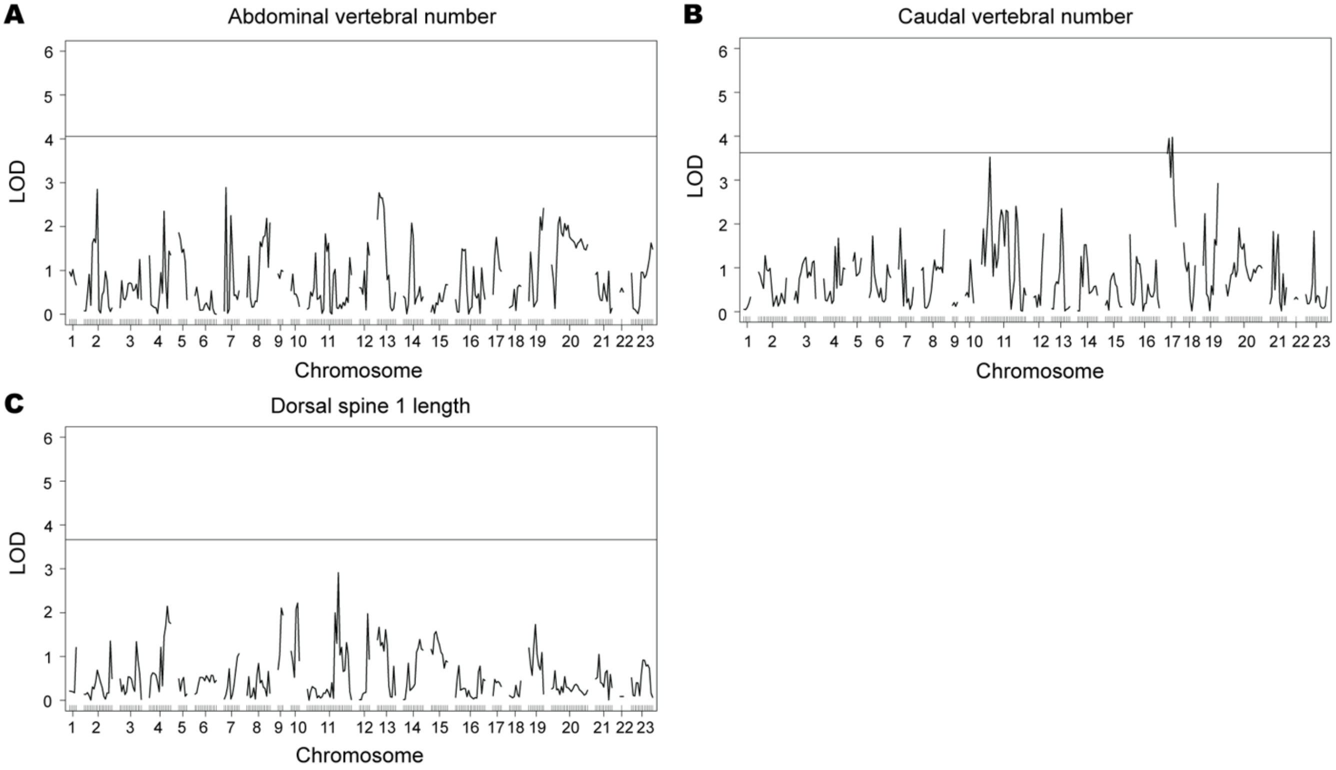

Supplemental Figure 1. Other axial traits had few or no significant QTL peaks.
Horizontal dotted lines in each graph represent genome-wide significance thresholds determined by 1,000 permutations of the data and α = 0.05.

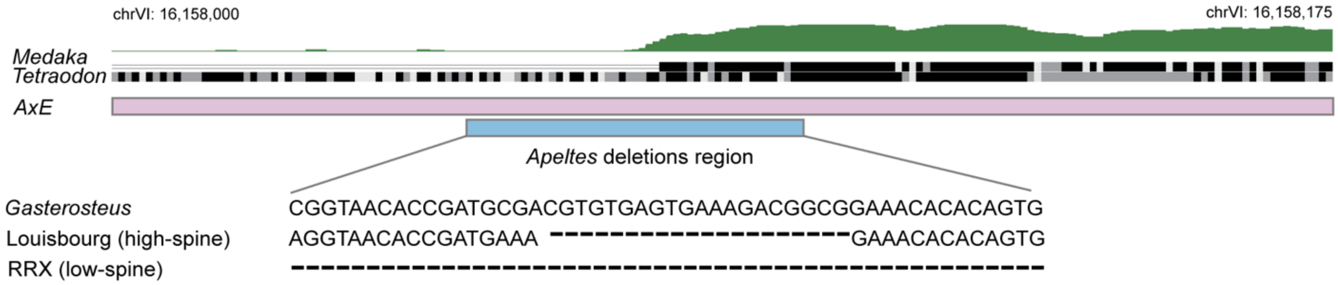

Supplemental Figure 2. Multi-species alignment of the *AxE* region. A multi-species alignment of a region of *AxE* shows two deletions (light blue region, dashes): a 20 bp deletion in Louisbourg *Apeltes* compared to *Gasterosteus* and a further 30 bp deletion in RRX *Apeltes* compared to Louisbourg. MultiZ track is shown for *Medaka* and *Tetraodon*. These deletions partially overlap a more broadly conserved region (green) between *Gasterosteus*, *Medaka*, and *Tetraodon*. Sequences are aligned to *Gasterosteus* (*gasAcu1*) in the UCSC genome browser (*Gasterosteus* bases from chrVI:16,158,050-16,158,099 shown).

Movie 1. A male *Apeltes* cares for a nest of embryos.

Movie 2. A male *Apeltes* cares for a nest of embryos built on an airstone.
